# Serrated Polyposis Syndrome: Increasing Awareness and Importance

**DOI:** 10.7759/cureus.9198

**Published:** 2020-07-15

**Authors:** Heeyah Song, Eula Tetangco, Elizabeth Martin, Dorian Willhite, John Erikson L Yap

**Affiliations:** 1 Medicine, Medical College of Georgia, Augusta, USA; 2 Divison of Gastroenterology and Hepatology, Medical College of Georgia at Augusta University, Augusta, USA; 3 Pathology, Medical College of Georgia, Augusta, USA; 4 Division of Gastroenterology and Hepatology, Medical College of Georgia at Augusta University, Augusta, USA

**Keywords:** sps, serrated polyposis syndrome, colorectal cancer

## Abstract

Serrated polyposis syndrome (SPS) was formerly considered a rare condition. In the past decade, it has gained increasing recognition due to its close association with colorectal cancer (CRC). Diagnosis is made based on the updated World Health Organization (WHO) criteria of having serrated polyps (SPs) proximal to the rectum, all being ≥5 mm in size, with at least two being ≥10 mm in size (criterion I), and a more distal phenotype that presents with greater than 20 SPs of any size throughout the large bowel with five being proximal to the rectum (criterion II). There are three subtypes of SP: hyperplastic polyp (HP), sessile serrated lesion (SSL), and traditional serrated adenoma (TSA). We present a 61-year-old Caucasian male who was referred for surveillance colonoscopy due to a history of colon polyps. A total of 28 polyps were completely removed, 21 of which were found to be SPs, three of which were >10 mm in size, meeting the WHO criteria for SPS. A follow-up colonoscopy was recommended in one year. It is now recognized that SPS are significant contributors to the development of CRC. The United States Multi-Society Preventive Task Force recently updated their consensus statement in 2020 with specific guidance for surveillance of SPs. It is important to emphasize that the diagnostic criteria apply to cumulative polyp count over the individual’s lifetime. The optimal surveillance for SPS remains unclear.

## Introduction

Serrated polyposis syndrome (SPS), previously known as hyperplastic polyposis syndrome, is a relatively new condition characterized by multiple serrated polyps (SPs) in the colon. From its first description in the literature in 1970s, its diagnostic criteria were established by Burt and Snover for WHO in 2010 [1-2]. Recent years have seen an increasing awareness of this entity due to its close association with colorectal cancer (CRC). The US Multi-Society Task Force on Colorectal Cancer recognizes SPS as a high-risk group along with other hereditary CRC syndromes and inflammatory bowel disease [3]. SPs are responsible for 25% of sporadic CRC [4]. With advancement in the quality of endoscopic procedures as well as increased awareness among the clinicians of the association of SPS with CRC, SPS has become the most common polyposis syndrome with its prevalence up to 1:111 colonoscopies in fecal immunochemical test (FIT)-based screening programs based on 2010’s diagnostic criteria [5]. We present the case of a 61-year-old male who was found to have multiple SPs of various subtypes on routine surveillance colonoscopy, fulfilling the criteria for SPS.

## Case presentation

**Figure 1:**
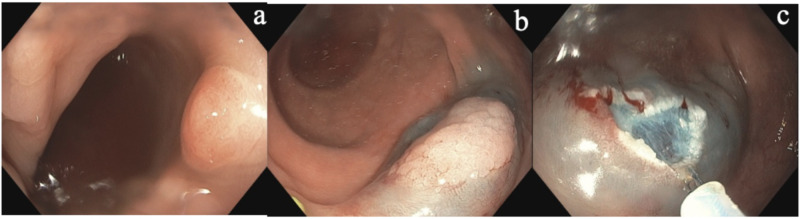
Representative (a) serrated lesion in the sigmoid colon that was (b) lifted and (c) removed by snare cautery.

**Figure 2:**
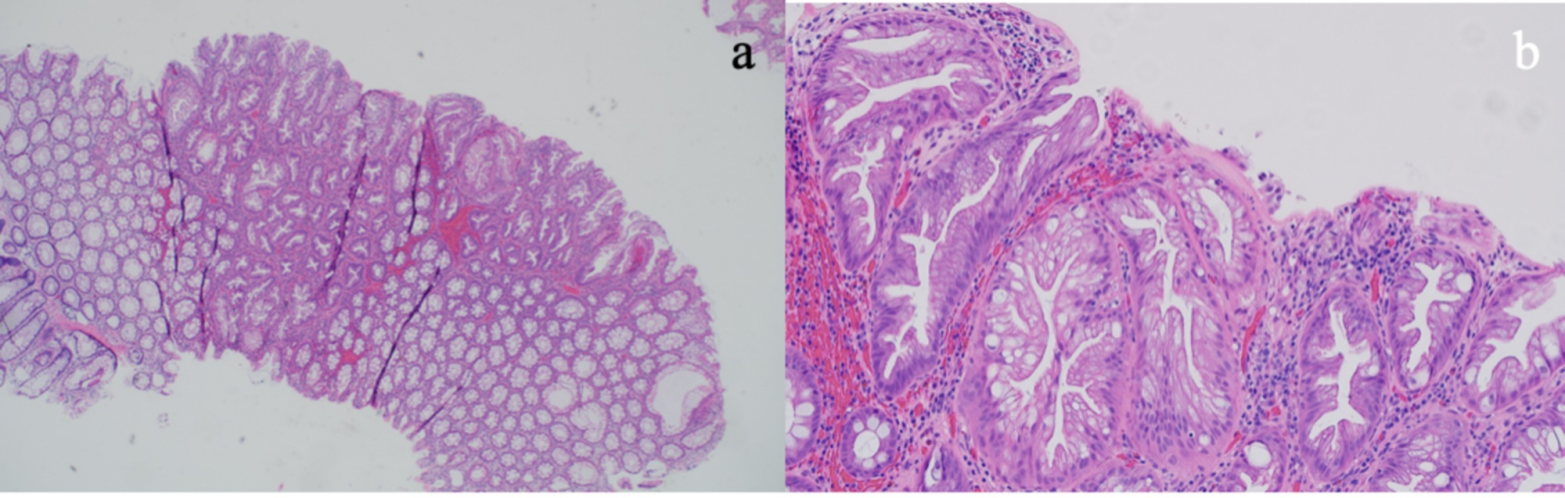
Histologic appearance of the serrated lesion in the sigmoid colon-hyperplastic polyp at 4x (a) and at 20x (b).

A 61-year-old Caucasian male with hypertension was referred to open access endoscopy for surveillance colonoscopy due to a history of colon polyps. His index colonoscopy nine years earlier showed two hyperplastic polyps (HPs), each measuring 3 mm, one in the cecum and one in the rectum. Physical examination was normal. Complete blood count and chemistries were unremarkable. He reported quitting smoking more than 20 years ago and denied any family history of colon cancer and SPS. Colonoscopy revealed a total of 28 polyps, which were completely removed (see Figure 1). Four polyps were seen in the cecum, two measuring 2 mm and two measuring 4 mm in size. Three polyps were seen in the ascending colon measuring 5 mm in size. One polyp was seen in the transverse colon measuring 2 mm in size. All of these were hyperplastic on pathology. Four polyps were seen in the descending colon. The first was a flat polyp measuring 10 mm in size that was removed via endoscopic mucosal resection (EMR). The other two polyps measured 4-5 mm in size. Pathology revealed these to be HPs and tubular adenomas. Four polyps were seen in the sigmoid colon. The first was a flat polyp measuring 15 mm, removed by EMR. The other three polyps measured 5 mm in size. Multiple sessile polyps were seen in the rectum measuring from 2 to 10 mm in size. Twelve total representative samples were taken. All sigmoid and rectal polyps were hyperplastic on pathology (Figure 2). Overall, out of the 28 total polyps removed, the patient had 21 SP, three of which were >10 mm in size. He was informed about his diagnosis of SPS. Surveillance colonoscopy is scheduled in one year.

## Discussion

Serrated polyps are common and are detected in 20% of all colonoscopies in average-risk subjects [6]. However, SPS as an entity is distinguished from the SP by the number, size, and location of these polyps. A patient is diagnosed with SPS if any of the World Health Organization (WHO) criteria are met. The recently updated 2019 WHO criteria for SPS recognize two types of the syndrome: SPs proximal to the rectum, all being ≥5 mm in size, with at least two being ≥10 mm in size (criterion I 2019), and a more distal phenotype that presents with greater than 20 SPs of any size throughout the large bowel (criterion II 2019) [7]. Importantly, any serrated polyp subtype is included in the polyp count, which is cumulative over multiple colonoscopies.

Serrated polyp is an umbrella term that refers to a polyp with “saw-tooth-like” appearance on histology [6]. It is further divided into three subtypes: HP, sessile serrated lesion (SSL), and traditional serrated adenoma (TSA). Although they share many histological features, each subtype has a distinct endoscopic appearance, molecular characteristics, and preferential location [4]. HPs are the most common subtype, accounting for ~70% of all SPs. These tend to occur in the distal colon [4]. On histology, HPs are further divided into two subtypes based on morphology: goblet cell-rich and microvesicular types. SSLs, which tend to occur in the proximal colon are identified by architectural distortion, predominantly crypt dilation and distortion in various forms [8]. TSAs are less common than HPs or SSLs and generally occur in the sigmoid and rectum. They are relatively larger than HP and SSL, and are identified histologically by hyperserration with ectopic crypt formation, eosinophilic cytoplasm, and villous pattern on histology. In general, HPs are deemed benign while SSLs and TSAs carry a higher risk of developing dysplasia and eventually progressing to CRC due to accumulation of molecular alterations [9].

It is estimated that 25%-70% of SPS patients develop CRC [10]. However, there has not been established guidelines for both screening and therapeutic management of SPS. A recent consensus update by the US Multi-Society Task Force in 2020 now recognizes the importance of SP in the pathogenesis of colon cancer. The guidelines recommend offering a follow-up colonoscopy to average risk patients based on number and size of SSL alone, but the diagnosis of SPS excludes patients from average risk patients [3]. Evidence suggests that SPS represents a range of multiple conditions with variable phenotypes and thereby variable risk of progressing to CRC [11]. A majority of the earlier studies on surveillance in SPS have been retrospective, with a few prospective cohort studies limited by short follow-up duration [12]. Subjecting all patients with SPS to a yearly colonoscopy surveillance as recommended by many international guidelines may seem like over-treatment for some patients, while a less rigorous approach poses the opposite problem of interval cancer. A recent cohort study of 142 patients with SPS were prospectively followed for over 10 years, with surveillance performed every one to two years. In up to nine rounds of surveillance, no upward or downward trend in polyp recurrence was observed. The authors therefore advocate for lifelong adherence to personalized surveillance guidelines, discouraging de-intensifying surveillance intervals. Hence, clinicians making the diagnosis of SPS must consider other aspects of the patient such as the individual polyp size, location, molecular pathology, family history, and other risk factors to adopt a more personalized approach. It is important to emphasize that the criteria apply to cumulative polyp count over the individual’s lifetime. This stresses the need to obtain prior colonoscopy and pathology reports for each patient.

This case underlies the challenges that clinicians diagnosing SPS faces as well as a need for more studies to investigate risk factors such that a more personalized approach to manage individual SPS can be developed.

## Conclusions

Here we present the case of a gentleman who on routine endoscopy had 28 polyps removed and was subsequently diagnosed with SPS. This case highlights the recent emergence of SPS, newly updated diagnostic criteria as well as its association with CRC. Given that SPS is strongly associated with CRC, lifelong surveillance is recommended but specific management approach should be personalized taking into account the polyp count, size, and other risk factors.
